# Optimization of Polyphenol Extraction Process from Yunnan-Grown Olives and Analysis of Taste Capacity Using Response Surface Methodology

**DOI:** 10.3390/molecules31162929

**Published:** 2026-08-21

**Authors:** Hongyang Pan, Zhaojun Wang, Jie Chen

**Affiliations:** 1 State Key Laboratory of Food Science and Resources, Jiangnan University, Wuxi 214122, China; rickypan@jiangnan.edu.cn (H.P.); zhaojun.wang@jiangnan.edu.cn (Z.W.); 2 Analysis and Testing Center, Jiangnan University, Wuxi 214122, China

**Keywords:** Yunnan-grown olives, polyphenols, extraction process optimization, response surface methodology (RSM), taste capacity, Central Composite Design (CCD)

## Abstract

This study aimed to optimise polyphenol extraction from Yunnan-grown olives (*Canarium album*), investigate key factors influencing taste capacity and their underlying mechanisms, and to provide technical support for industrial scale-up. Initial single-factor experiments assessed the effects of seven operational parameters on extraction yield and sensory quality. Subsequently, ethanol concentration, extraction temperature, extraction time, and pH were selected as independent variables to optimize the process and validate the model via a Central Composite Design (CCD), using total polyphenol extraction yield and taste capacity as response values. The results showed that single-factor experiments identified 60% ethanol as the optimal solvent, with enhanced extraction efficiency at a 3 h extraction time, a temperature of 50 °C, and a pH of 3. Quantitative analysis during single-factor experiments revealed strong correlations between extraction yield and taste capacity (r > 0.88). The response surface model exhibited a high goodness-of-fit; the impact of individual factors on the extraction yield followed the order of extraction temperature > extraction time > ethanol concentration > pH, whereas the order for taste capacity was extraction temperature > extraction time > pH > ethanol concentration. Under optimal process conditions, the total polyphenol extraction yield reached 216.38 ± 3.60 mg/g, with a taste capacity of 1.27 ± 0.02, showing no significant difference between the predicted and experimental values (*p* > 0.05). By synergistically regulating extraction parameters, the optimized process successfully balances efficient polyphenol dissolution with desirable taste characteristics. These findings provide a solid scientific reference for the value-added development of Yunnan-grown olive polyphenols.

## 1. Introduction

Olives [*Canarium album* (Lour.) Raeusch], a characteristic tropical and subtropical fruit tree in China, produce fruit rich in polyphenolic compounds. These compounds exhibit antioxidant, hepatoprotective, and anti-inflammatory activities and have broad application prospects across the food, pharmaceutical, and healthcare industries [1]. Due to their unique eco-geographical environment (characterized by high altitude and intense ultraviolet radiation), Yunnan-grown olives exhibit a distinct phenolic composition. While sharing some common polyphenols, they are particularly rich in secoiridoids, such as hydroxytyrosol and its glucosides, which are well-recognized for their potent bioactivities. This profile differs markedly from that of Mediterranean olives (*Olea europaea*), which are predominantly studied for their oleuropein and related aglycones. Furthermore, the high-altitude, high-UV environment of Yunnan appears to enrich specific hydrolyzable tannins and phenolic acids, contributing to their unique functional properties. However, their industrial development is largely constrained by the need for scientific and efficient extraction methods [2,3]. The extraction efficiency of plant polyphenols depends not only on the characteristics of the raw materials but also on the extraction methods and process parameters. Therefore, optimizing the extraction process to enhance polyphenol yield and quality has become a research hotspot in this field [4,5]. Moreover, modern extraction methodologies increasingly rely on miniaturized and modified techniques, such as QuEChERS combined with chromatography, to maximize the recovery of active ingredients from complex food matrices [6].

Traditional solvent extraction remains the core technique for plant polyphenols, relying on differential solubility across various solvent media [7,8,9]. Early studies primarily focused on screening single solvents, ranging from water, methanol, and ethanol to acetone, and gradually established that complex solvent systems (with organic solvent proportions of 50–70%) perform significantly better than single solvents by disrupting hydrogen and hydrophobic bonds between polyphenols and proteins or polysaccharides [10,11,12]. As research deepened, scholars found that parameters such as extraction time, temperature, pH, liquid-to-solid ratio, and the number of extractions all significantly affect polyphenol dissolution. Although single-factor optimization can initially determine the suitable range for each parameter, it fails to reveal interactions among factors, thereby limiting process optimization [13,14]. Particularly in challenging matrices, comprehensive sample preparation methods are indispensable to mitigate matrix effects prior to chromatographic evaluation [15].

To address multi-factor interactions, process optimisation methods have gradually shifted from single-factor experiments to multivariate statistical optimisation. As an early multi-factor optimisation tool, orthogonal experimental design identified optimal parameter combinations within a limited number of trials through carefully arranged experimental designs. However, this method only examines linear relationships and struggles to accurately capture nonlinear patterns between parameters and response values. The emergence of Response Surface Methodology (RSM) provided a new pathway for optimising complex processes. Based on designs such as the Central Composite Design (CCD), RSM builds quadratic regression equations to systematically analyse how multi-factor interactions affect response values, thereby enabling precise parameter prediction and optimisation [16,17,18]. RSM has been widely used to optimise the extraction of natural products, including tea, grape polyphenols, and blueberry polyphenols. Compared with traditional methods, it offers high experimental efficiency, superior optimisation accuracy, and the ability to establish mathematical models, making it the mainstream approach for optimising the extraction of plant active ingredients [19,20,21].

Despite significant progress in polyphenol extraction research, studies on polyphenols from Yunnan-grown olives still exhibit several deficiencies: First, existing research mostly focuses on a single extraction rate indicator, neglecting the correlation between polyphenol content and product taste characteristics, making it difficult to meet the dual demands of functionality and sensory quality in the food and tobacco industries [22,23,24]. To bridge this gap, this study introduces the concept of “taste capacity,” defined as the ratio of a specific compound’s concentration to its taste threshold, which quantitatively evaluates the impact of extracted constituents on sensory perception. Based on previous studies, only compounds with a “Dot value” (concentration/threshold) greater than 1 significantly contribute to the overall taste profile. In sensory science, the overall perception of a complex mixture is not merely the sum of its individual parts but is governed by complex interactions such as synergy, masking, and additive effects. To provide a quantitative and reproducible measure of these sensory interactions, we adopt a pragmatic approach based on the “Dot value” (concentration/threshold ratio), a well-established metric in flavor chemistry. The taste capacity of an extract is operationally defined as the aggregate sum of the Dot values for its major quantified polyphenolic constituents (e.g., hydroxytyrosol, gallic acid, specific flavonoids). According to this definition, only compounds with a Dot value > 1 are considered to contribute significantly to the overall sensory profile. This integrative index serves as a crucial link between the chemical composition of the extract and its potential sensory impact, making the optimization process directly relevant for applications in the food and tobacco industries where both functionality and consumer acceptance are paramount. Second, there is a lack of research on multi-factor interactions specifically targeting Yunnan-grown olives, resulting in a lack of systematic optimization of process parameters [25]. Third, some optimization methods used in previous studies lack precision and fail to develop reliable mathematical models for industrial production prediction [26,27]. Therefore, integrating precise multi-factor DOE modeling with robust sensory-chemical correlation analyses is essential to systematically elevate the quality standards of specialized agricultural products.

Building on the current research status, this study uses Yunnan-grown olives as raw materials. Unlike conventional studies that merely adjust common extraction parameters for maximum yield, this dual-optimization strategy represents a novel approach to resolving the conflict between functional ingredient concentration and sensory acceptability. Drawing on advances in plant polyphenol extraction methods, we adopted a classic research paradigm of “single-factor preliminary experiments combined with RSM optimization”. First, single-factor experiments evaluated the effects of seven key factors on the polyphenol extraction rate, identifying suitable ranges for each parameter. Next, key factors were selected to build a quadratic regression model using a five-level Central Composite Design (CCD), with total polyphenol extraction rate and taste capacity as dual response variables. This systematic analysis of factor interactions aimed to determine the optimal extraction process. This study not only provides technical support for the industrial extraction of polyphenols from Yunnan-grown olives but also offers a methodological reference for the multi-objective optimization of natural product extraction processes, further enriching the application of RSM in the deep processing of specialty agricultural products.

## 2. Results and Discussion

### 2.1. Single-Factor Experiment Results

**Figure 1:**
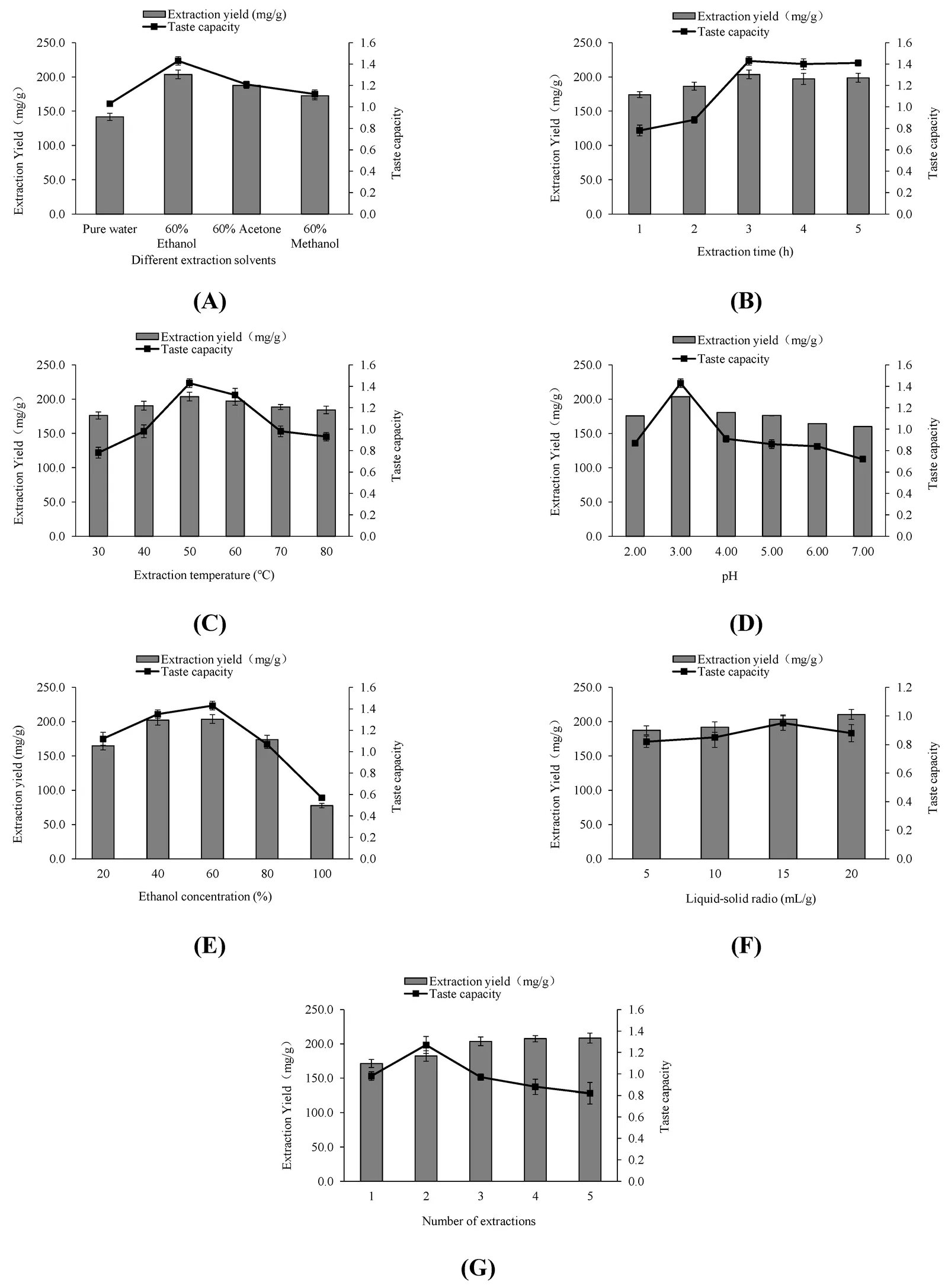
Effects of different single-factor extraction parameters on the total polyphenol extraction yield and taste capacity of Yunnan-grown olives: (**A**) solvent type; (**B**) extraction time; (**C**) extraction temperature; (**D**) pH; (**E**) ethanol concentration; (**F**) liquid-to-solid ratio; (**G**) extraction frequency.

To systematically evaluate individual factors, standard baseline conditions were established as follows: 60% ethanol, 3 h extraction time, 50 °C, pH 3, 15 mL/g liquid-to-solid ratio, and 3 extraction cycles. When testing a specific variable, all other parameters were strictly held constant at these baseline values. To streamline the presentation and facilitate data interpretation, all graphical data from the single-factor experiments have been combined into Figure 1A–G, and their core statistical parameters are summarized in Table 1.

#### 2.1.1. Effect of Different Solvents on Extraction Efficiency

The extraction yield of polyphenols via solvent extraction depends on the dissolution state of individual phenolic compounds within plant cells and on their diffusion behavior into the external solvent. Since polyphenols typically form stable molecular complexes with proteins and polysaccharides in plants via hydrogen bonds and hydrophobic interactions, the extraction reagent must not only exhibit excellent solubility for polyphenols but also be capable of disrupting hydrogen bonds [18,20]. Consequently, binary solvent systems consisting of an organic solvent and water (with organic solvent ratios of 50–70%) are most suitable for polyphenol extraction.

The objectives of solvent screening are, on the one hand, to evaluate the extraction efficiency of various solvents and, on the other hand, to identify safe alternatives to toxic traditional extraction solvents, ensuring the safe application of functional ingredients in the tobacco, food, and pharmaceutical industries [11,12]. In parallel, emerging green microextraction strategies, including those utilizing in situ generated dendritic ionic liquids, have also demonstrated exceptional selectivity and efficiency for target compounds without the use of dispersive organic solvents [28].

When using water, 60% ethanol, 60% methanol, and 60% acetone solutions with varying polarities to extract Yunnan-grown olive powder, significant differences were observed in both the total polyphenol extraction yield and the taste capacity of the extracts (*p* < 0.05).

Among the tested solvents, water yielded the lowest polyphenol extraction (141.8 ± 5.2 mg/g) and taste capacity (1.03 ± 0.02), whereas 60% ethanol demonstrated the highest efficiency (203.7 ± 6.3 mg/g and 1.43 ± 0.04, respectively) (Figure 1A). A significant positive correlation was observed between polyphenol extraction yield and taste capacity across solvents (Pearson’s r=0.92, p<0.05). Therefore, ethanol was selected as the extraction solvent to optimize the process.

#### 2.1.2. Effect of Extraction Time on Extraction Efficiency

Extraction time affects the contact between the solvent and active substances located in different parts of the cells, governing their dissolution and release. Prolonged extraction allows the solvent to fully penetrate various parts of Yunnan-grown olive tissues, ensuring sufficient contact and dissolution of active ingredients, thereby increasing the extraction yield; however, excessively long extraction times can cause the oxidative degradation of phenolic substances [8,17].

As shown in Figure 1B, the extraction yield increased rapidly within the first 3 h, after which mass transfer equilibrium was reached, resulting in no further significant gains at 4 h. The taste capacity of the extract obtained at 3 h was significantly higher than those at 1 h and 2 h (*p* < 0.05). No significant changes in taste capacity were observed when the extraction time exceeded 3 h. A significant correlation was found between polyphenol extraction yield and taste capacity across different extraction times (Pearson’s *r* = 0.949, p<0.05). Therefore, an extraction time of approximately 3 h was chosen.

#### 2.1.3. Effect of Extraction Temperature on Extraction Efficiency

Extraction temperature is a critical parameter that influences extraction efficiency. It has been reported that extracting polyphenols from fruits at higher temperatures can increase product yields, potentially because high-temperature solvents facilitate the dispersion of cell-wall polysaccharides into the solvent system, weakening or disrupting cell-wall integrity and enabling better contact between the solvent and the polyphenols, thereby enhancing extraction yield [7,9].

As illustrated in Figure 1C, the total polyphenol extraction yield increased with temperature up to 50 °C. Temperatures exceeding 50 °C caused a sharp decline in yield, likely due to the thermally induced oxidative degradation and denaturation of phenolic compounds, which concurrently reduced the taste capacity. A strong correlation was observed between polyphenol extraction yield and taste capacity across extraction temperatures (Pearson’s *r* = 0.966, p<0.01). Therefore, an extraction temperature of around 50 °C is advisable.

#### 2.1.4. Effect of pH on Extraction Efficiency

Figure 1D demonstrates that optimal extraction occurred at a pH of approximately 3, yielding 203.7 ± 6.3 mg/g. Under acidic conditions, polyphenols form oxonium salts that ionize readily, dissolve, and elute. Furthermore, polyphenol oxidase activity is suppressed in acidic environments, thereby inhibiting the enzymatic oxidation of polyphenols during extraction. Additionally, polyphenols are less sensitive to oxygen and light under acidic conditions, reducing oxidative degradation and consequently increasing the total polyphenol extraction yield [18,24]. Therefore, pH 3 was selected for further experiments.

#### 2.1.5. Effect of Ethanol Concentration on Extraction Efficiency

The distribution of polyphenolic substances within plant tissues varies at the cellular level. Soluble phenolics are primarily located in cell vacuoles, whereas lignin, flavonoids, and insoluble polyphenols are mostly deposited in cell walls, bound to proteins and polysaccharides via hydrogen bonds and hydrophobic interactions. Water and low-concentration ethanol can freely pass in and out of cells, but high-concentration ethanol can denature proteins within tissues, thereby hindering polyphenol dissolution and compromising extraction yield.

Although water is an effective solvent for polyphenols, it cannot disrupt hydrogen bonds. The addition of ethanol disrupts these bonds, facilitating the dissolution of phenolic substances and thus enhancing the extraction yield [20]. Varying ethanol concentrations alter the polarity of the solvent system, and an optimal concentration exists that maximizes the polyphenol extraction yield. The effects of different ethanol concentrations on the polyphenol extraction efficiency of Yunnan-grown olives are shown in Figure 1E.

60% ethanol achieved the highest extraction efficiency for Yunnan-grown olive polyphenols, yielding 203.7 ± 6.3 mg/g. Ethanol concentration significantly affected the taste capacity of the extracts (*p* < 0.05), with the peak capacity also observed at 60% ethanol (1.43 ± 0.04). A significant correlation was observed between polyphenol extraction yield and taste capacity across ethanol concentrations (Pearson’s r=0.91, p<0.05). Consequently, an ethanol concentration of approximately 60% was selected as the extraction solvent for further single-factor baseline optimization.

#### 2.1.6. Effect of Liquid-to-Solid Ratio on Extraction Efficiency

Aqueous solutions of organic solvents can cleave the bonds among polyphenols as well as those between polyphenols and proteins or polysaccharides, favoring the liberation of phenolics. The liquid-to-solid ratio governs the dissolution status of the extraction solvent and phenolic substances. As the liquid-to-solid ratio increases, polyphenol extraction yield increases; however, an excessively high liquid-to-solid ratio results in waste of solvent and energy. Increasing the liquid-to-solid ratio up to 15 mL/g improved the yield, beyond which mass transfer equilibrium was reached, resulting in no further significant gains (Figure 1F). The taste capacity at this baseline point was 0.95 ± 0.05. Furthermore, changes in the liquid-to-solid ratio had no significant linear correlation with the taste capacity (Pearson’s *r* = 0.689, *p* > 0.05).

#### 2.1.7. Effect of Extraction Frequency on Extraction Efficiency

The results of this experiment (Figure 1G) showed that the extraction yield of Yunnan-grown olive polyphenols increased with higher extraction frequency. Three extractions yielded 203.7 ± 6.3 mg/g, significantly higher than the 182.3 ± 7.6 mg/g obtained from two extractions (*p* < 0.05).

The extraction yield after four extractions was 207.6 ± 4.5 mg/g, which did not differ significantly from that after three extractions. However, after three extraction cycles, the taste capacity of the resulting polyphenol extract decreased significantly (*p* < 0.05). While taste capacity decreased at three cycles, this frequency was purposely selected to ensure that the maximum total polyphenol yield was achieved as a foundation for the subsequent response surface modeling. This approach guarantees that the model has an adequate upper limit of yield from which to compute the optimal compromise between extraction efficiency and sensory quality. A three-cycle extraction scheme was deemed appropriate.

### 2.2. Response Surface Analysis and Process Optimization

#### 2.2.1. Establishment of the Quadratic Regression Equation Model and Process Optimization

Based on the principles of the Box–Behnken central composite experimental design and single-factor experimental results, four factors that significantly affect the total polyphenol extraction yield and taste capacity—ethanol concentration, extraction temperature, extraction time, and pH—were selected for a response surface analysis consisting of 30 experimental runs across 4 factors and 5 levels. The center-point experiment was repeated six times to improve the sensitivity and accuracy of experimental error estimation. The experimental factors and levels are presented in Table 1, and the experimental layout and corresponding results are summarized in Table 2.

The response values were fitted to a quadratic polynomial equation using the least-squares method: $Y=A_{0}+\sum A_{i}X_{i}+\sum A_{ii}X_{i}^{2}+\sum A_{ij}X_{i}X_{j}$, where $Y_{1}$ is the response value for extraction yield; $Y_{2}$ is the response value for taste capacity; $A_{0}$, $A_{i}$, $A_{ii}$, and $A_{ij}$ are the polynomial coefficients; and $X_{i}$ and $X_{j}$ (i≠j) represent the coded values of the independent variables. Statistical F-values, p-values, and analysis of variance (ANOVA) were employed to evaluate the fit and regression of individual coefficients in the model equations.

Using the design matrix generated via the Central Composite Design (CCD) (detailed in Materials and Methods Section 3.4), the response values for extraction yield (Y_1_) and taste capacity (Y_2_) from the 30 experimental runs were analyzed. The experimental layout and observed response values are given in Table 3. To enhance data interpretability, the following equations utilize actual input values for the experimental factors rather than coded variables. Here, X_1_, X_2_, X_3_, and X_4_ represent the actual values for ethanol concentration (%), extraction temperature (°C), extraction time (h), and pH, respectively:

(1) $$\begin{array}{cc} Total\;Polyphenol\;Extraction\;Yield\;(mg/g)\;=\; & 213.13+2.13X_{1}-4.65X_{2}+3.07X_{3}+0.04X_{4}\\ & -1.93X_{1}X_{2}-0.71X_{1}X_{3}+2.61X_{1}X_{4}-1.09X_{2}X_{3}\\ & +2.09X_{2}X_{4}-0.76X_{3}X_{4}-0.58X_{1}^{2}-4.88X_{2}^{2}\\ & -4.48X_{3}^{2}-0.79X_{4}^{2} \end{array}$$

(2) $$\begin{array}{cc} Taste\;Capacity\;=\; & 1.202+0.018X_{1}-0.051X_{2}+0.041X_{3}+0.020X_{4}\\ & -0.013X_{1}X_{2}-0.003X_{1}X_{3}+0.031X_{1}X_{4}-0.030X_{2}X_{3}\\ & +0.021X_{2}X_{4}-0.019X_{3}X_{4}-0.015X_{1}^{2}-0.080X_{2}^{2}\\ & -0.083X_{3}^{2}-0.023X_{4}^{2} \end{array}$$

Discussion of the regression equations reveals the complex nonlinear relationships governing the extraction. For instance, in Equation (1), the large negative coefficients for the quadratic terms (e.g., −4.88 for $X_{2}^{2}$) confirm a strong parabolic curvature, indicating distinct optimal peaks within the tested ranges rather than linear trends.

**Figure 2:**
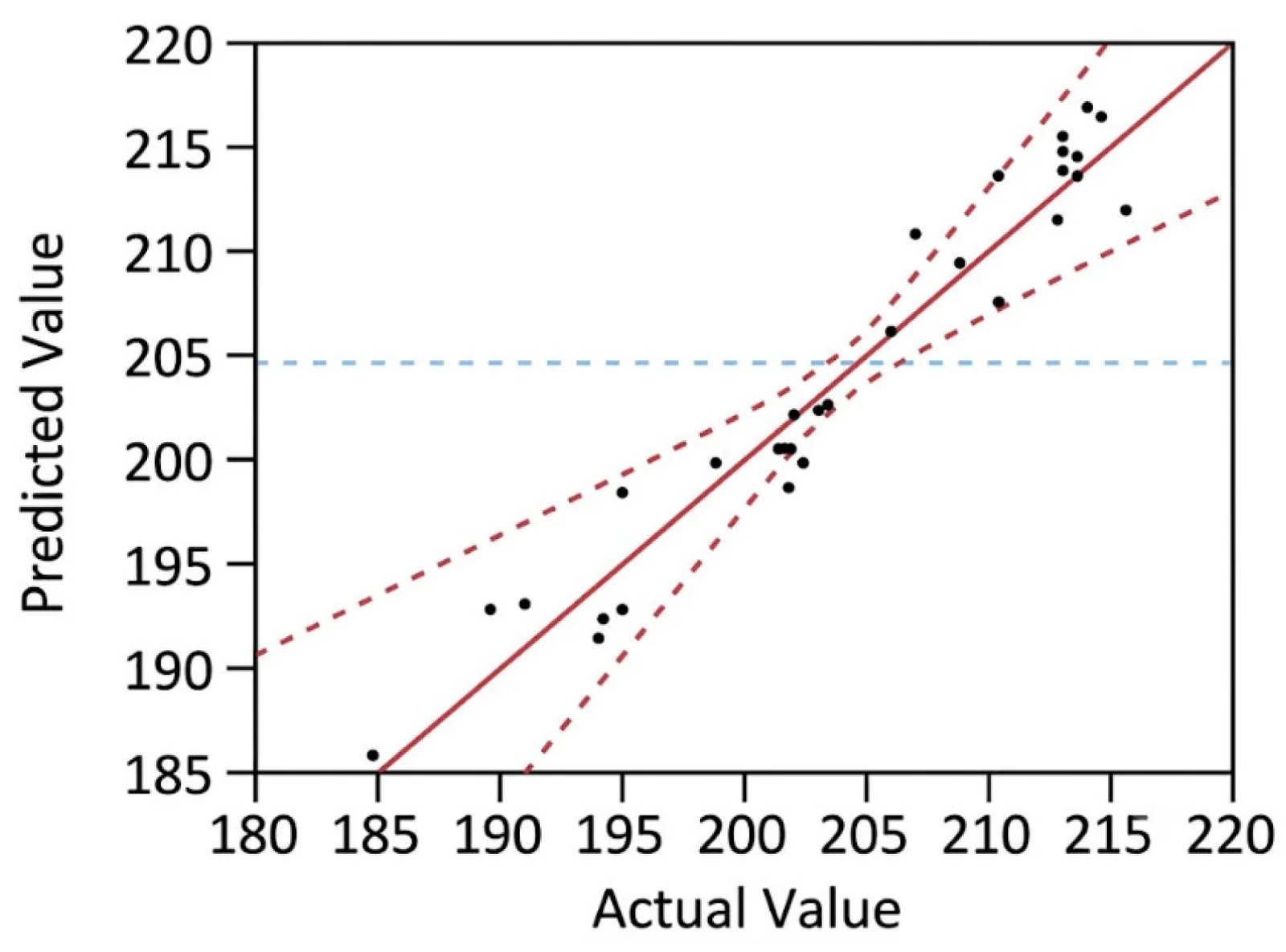
Comparison of predicted and experimental polyphenol extraction yields from Yunnan-grown olives. The red solid line represents the ideal 1:1 fit line, red dotted lines denote the confidence interval, and the blue dotted line indicates the mean value.

**Figure 3:**
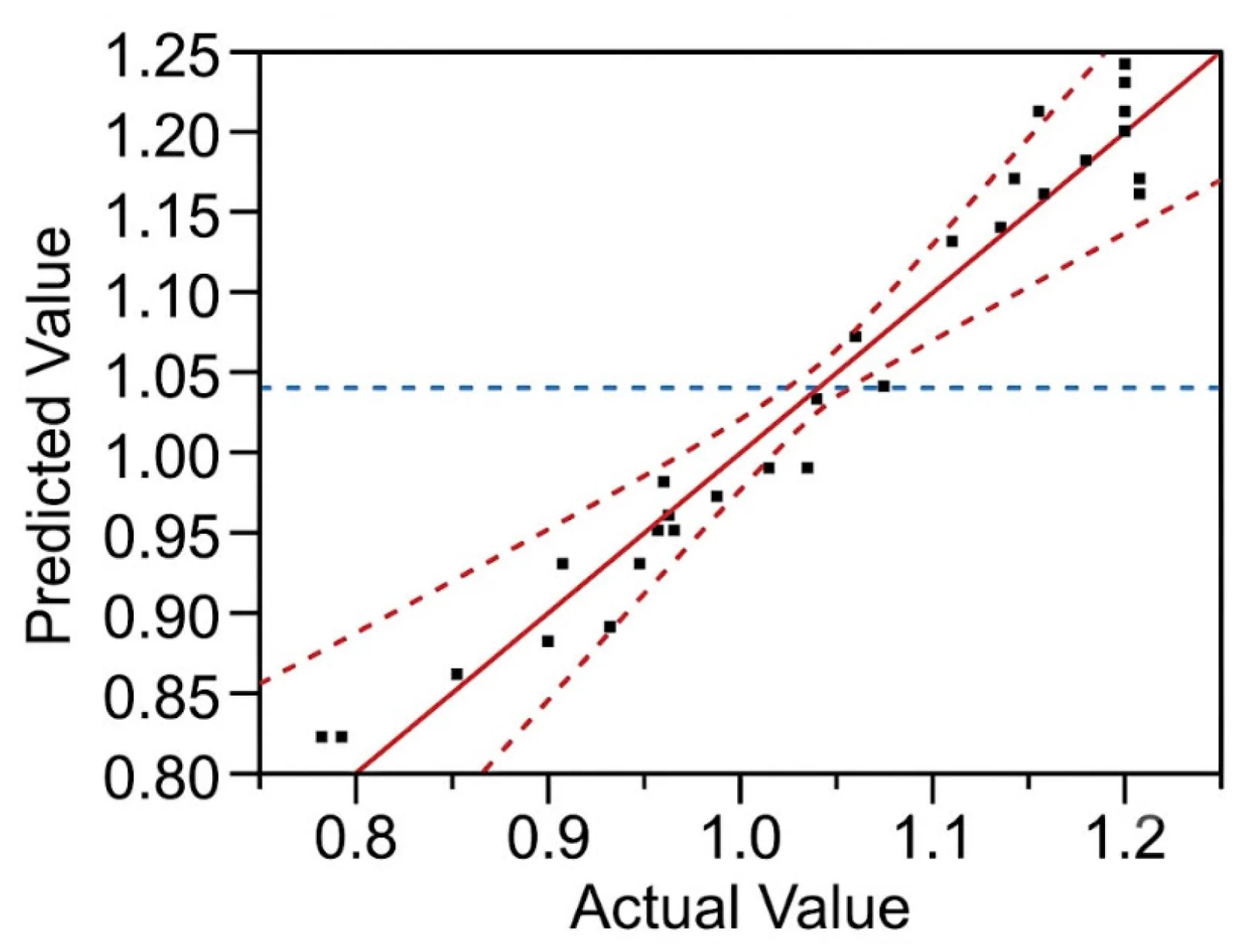
Comparison of predicted and experimental values for the taste capacity of Yunnan-grown olive polyphenols. The red solid line represents the ideal 1:1 fit line, red dotted lines denote the confidence interval, and the blue dotted line indicates the mean value.

ANOVA (Table 4 and Table 5) showed that the multiple regression relationships between the dependent variables and all independent variables, as described by Equations (1) and (2), were highly significant. The coefficients of determination (R^2^) were 0.9433 and 0.9595, and the model F-values were 13.5226 and 19.2637, respectively. Both F-values far exceeded the critical F_0.01_ values (14, 15), indicating extremely significant results. This confirms that the experimental design was reasonable and that the regression equations fit the experimental data well within the tested domain. Concurrently, the lack-of-fit tests for both models were not significant, indicating that Equations (1) and (2) fit well across the entire regression region. The predicted versus experimental values for polyphenol extraction yield and taste capacity are shown in Figure 2 and Figure 3, respectively. The data points cluster tightly along the 45-degree reference line, further confirming the high predictive reliability and precision of the established models across the designated design space.

The ANOVA results of the regression model for polyphenol extraction yield are summarized in Table 4. The corresponding ANOVA results for the taste-capacity regression model are shown in Table 5.

Based on the F-values, the order of importance of the factors influencing polyphenol extraction yield was: extraction temperature > extraction time > ethanol concentration > pH. For taste capacity, the order of influence was: extraction temperature > extraction time > pH > ethanol concentration.

The desirability criteria for the overall optimization were defined to simultaneously maximize both the total polyphenol extraction yield and the taste capacity, assigning equal weighting to both responses to ensure a balanced functional and sensory profile. Parameter optimization using the established model to maximize total polyphenol extraction yield identified the optimal coded parameters: X_1_ = 0.0386, X_2_ = −0.8149, X_3_ = 0.5456, and X_4_ = −1.2558. Converting these to actual values gave the optimal process conditions: ethanol concentration of 55.19%, extraction temperature of 50.93 °C, extraction time of 3.77 h, and pH of 2.37. It should be noted that while single-factor experiments identified 60% ethanol as optimal, the RSM optimization shifted this to 55.19%. This difference highlights the advantage of RSM in capturing multi-factor synergies; specifically, the highly optimized temperature and pH environment reduced the reliance on higher ethanol concentrations to successfully disrupt the hydrogen bonds in the plant matrix. For highly complex extracts, innovative clean-up techniques, such as PRiME pass-through procedures, have also proven highly effective for interference removal prior to high-resolution quantification [29]. Under these optimized conditions, the predicted polyphenol extraction yield was 215.57 mg/g. To physically verify the significance of the model, an actual verification extract was prepared under these exact optimized criteria. The actual experimental value obtained was 216.38 ± 3.60 mg/g for yield, and the taste capacity was 1.27 ± 0.02, showing no significant difference from the predicted values (*p* > 0.05). Comparing this yield with published reports on *Canarium album*, which typically report yields ranging from 120 to 160 mg/g under standard solvent extraction without RSM optimization, our optimized protocol demonstrates a substantial 35–80% improvement in extraction efficiency [7,25,27].

Parameter optimization to maximize taste capacity yielded the following coded parameters: X_1_ = 3.3227, X_2_ = −0.2156, X_3_ = −0.0619, and X_4_ = 2.6272. The corresponding actual conditions were: ethanol concentration of 71.61%, extraction temperature of 56.08 °C, extraction time of 3.47 h, and pH of 4.31. Under this taste-optimized process, the predicted taste capacity was 1.26, and the experimental value was 1.27.

Evaluating the taste capacity of the product obtained via the yield-optimized process (55.19% ethanol, 50.93 °C, 3.77 h, pH 2.37) yielded an experimental value of 1.27, which was not significantly different from the actual value (1.27) obtained under the taste-optimized process. Consequently, subsequent experiments focused primarily on the process optimized for total polyphenol extraction yield.

To verify the reliability of the optimization approach, verification experiments were conducted using the optimized parameter combination. The experimental total polyphenol extraction yield was 216.38 ± 3.60 mg/g, and the taste capacity was 1.27 ± 0.02. At a significance level of *p* < 0.05, no significant differences were observed between the predicted and experimental values. Thus, the extraction parameters optimized via response surface methodology are accurate, reliable, and practically valuable.

The successful integration of “taste capacity” as a dual-response variable alongside extraction yield provides a nuanced perspective on process optimization. The high correlation (r > 0.88) observed between yield and taste capacity in single-factor experiments suggests that, under certain conditions, enhanced polyphenol dissolution directly translates to a more intense sensory experience. However, the divergence in the order of factor influence for yield versus taste capacity indicates that this relationship is not always linear, particularly under extreme conditions, where oxidative degradation may preferentially affect compounds with lower taste thresholds, thereby altering the sensory profile independent of the total yield. This finding underscores the value of the taste capacity metric: it acts as a safeguard, ensuring that the optimization process does not merely maximize quantity at the expense of sensory quality. By balancing these two responses, the optimized process successfully yields an extract that is both functionally potent and sensorially acceptable, which is a critical consideration for its eventual industrial application.

While the established conditions provide excellent yields, industrial applicability and economic feasibility must be carefully considered. Maintaining the optimal extraction temperature within a narrow range (approximately 50–51 °C) is critical to preventing thermal degradation of polyphenols; on an industrial scale, this requires the use of precision-jacketed reactors or continuous-flow automated heating systems. Additionally, while the strongly acidic medium (pH 2.37) successfully prevents polyphenol oxidase activity, it dictates the use of acid-resistant manufacturing equipment (e.g., 316 L stainless steel or glass-lined tanks) and requires a neutralization step using food-grade bases prior to final product formulation to ensure the stability of other acid-sensitive constituents. Furthermore, although ethanol is a relatively GRAS and recoverable solvent compared to acetone or methanol, future scale-up protocols would benefit from comprehensive greenness assessments (e.g., AGREE or EcoScale) to formally quantify the environmental impact of the entire extraction and neutralization cycle [12].

#### 2.2.2. Interaction Effects Among Factors

**Figure 4:**
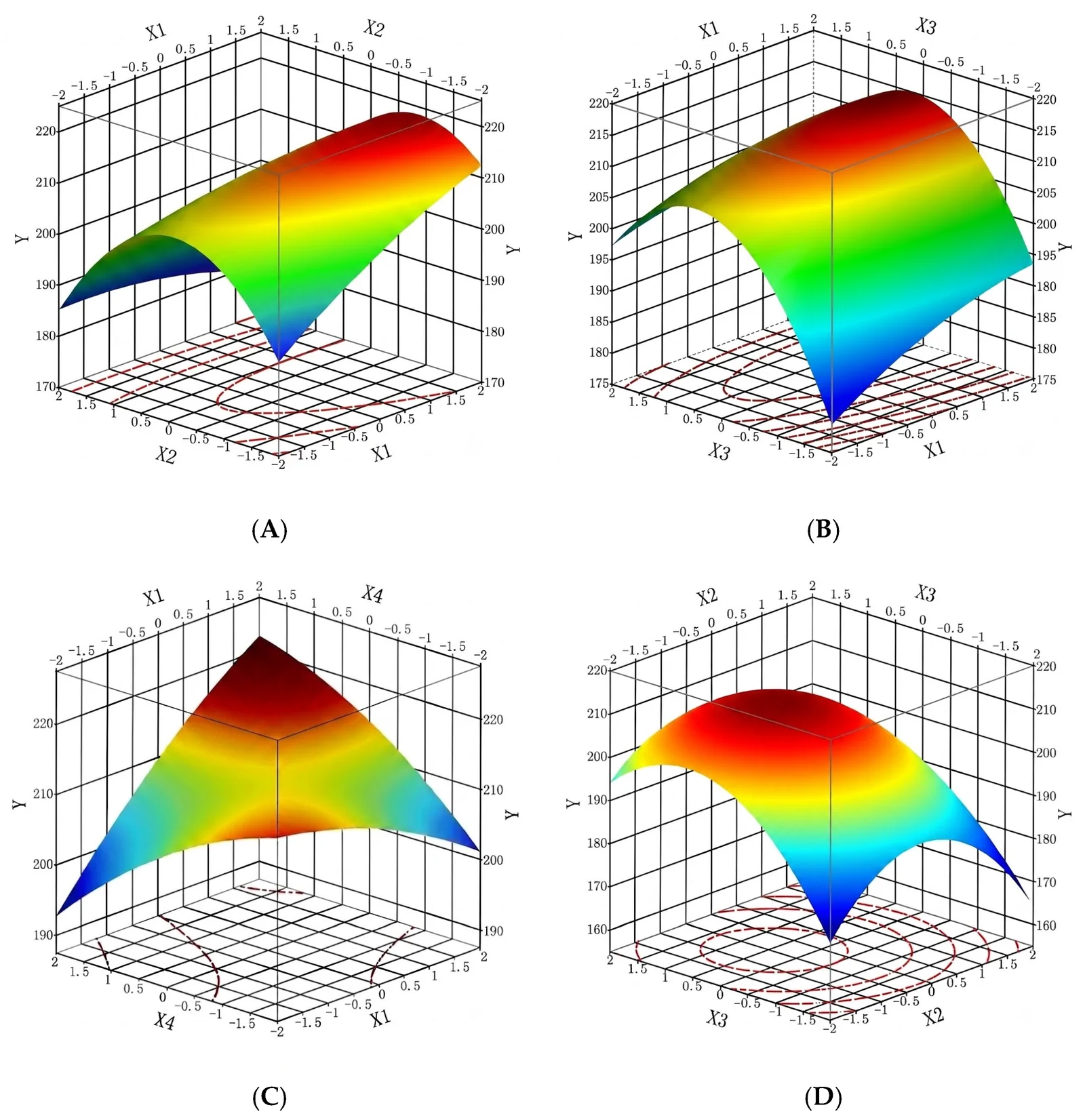

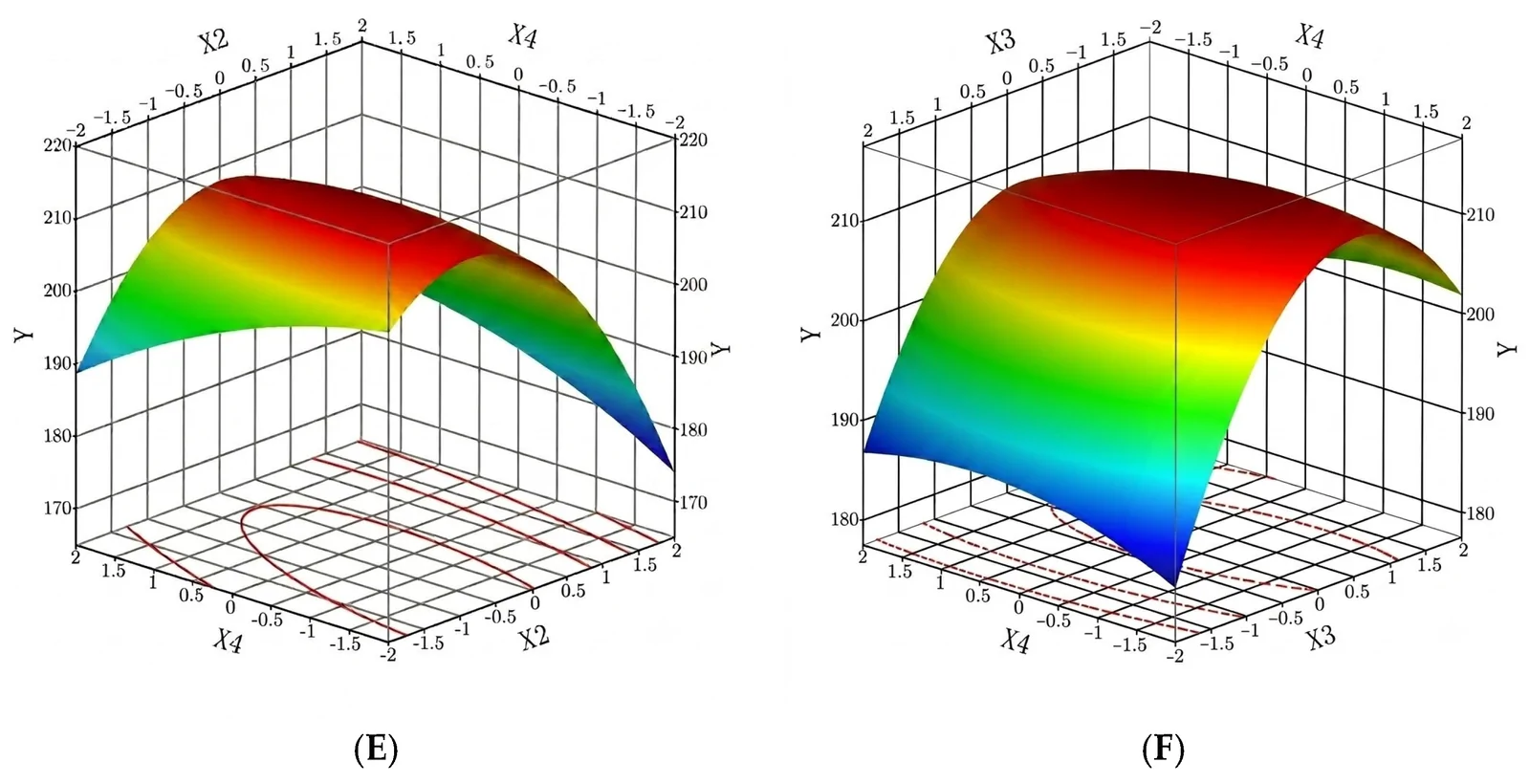
Three-dimensional response surface plots showing the interaction effects of independent variables on the polyphenol extraction yield: (**A**) ethanol concentration and extraction temperature; (**B**) ethanol concentration and extraction time; (**C**) ethanol concentration and pH; (**D**) extraction temperature and extraction time; (**E**) extraction temperature and pH; (**F**) extraction time and pH.

To facilitate interpretation, the coded values have been replaced with actual input parameters in the 3D response surface plots (Figure 4A–F), which have been grouped together and rendered in color to distinctly highlight the optimal response areas. Interaction effects among parameters can be clearly deduced from these plots.

As shown in Figure 4A, at lower extraction temperatures, the polyphenol extraction yield increased gradually with rising temperature and higher ethanol concentrations within a specific range. Mechanistically, this interaction occurs because moderate heat weakens the rigid plant cell walls and facilitates solvent penetration, thereby reducing the demand for extremely high ethanol concentrations to disrupt the hydrophobic and hydrogen bonds linking polyphenols to structural proteins. However, when the extraction temperature exceeded a threshold, the polyphenol yield declined despite further increases in ethanol concentration. This phenomenon can be attributed to accelerated oxidative degradation and denaturation of phenolic compounds at elevated temperatures, resulting in a loss of total polyphenols.

As shown in Figure 4B, both increasing the ethanol concentration and extending the extraction time increased the polyphenol extraction yield, with extraction time having a more pronounced effect than ethanol concentration. To achieve the same polyphenol extraction yield, a higher ethanol concentration allowed for a shorter extraction time, and vice versa.

Figure 4C shows that at a constant ethanol concentration, the polyphenol extraction yield decreased as pH increased from 2.50 to 3.50. At a fixed pH, the extraction yield increased with ethanol concentration. Under highly acidic conditions, polyphenols readily form more stable oxonium salts, mitigating the need for maximum ethanol polarity. At higher pH levels, the polyphenol extraction yield increased significantly with ethanol concentration, whereas at lower pH levels, changes in ethanol concentration had a negligible effect on the yield.

Figure 4D shows that at a fixed extraction time, polyphenol extraction yield decreased as extraction temperature increased. At lower extraction temperatures, extending the extraction time from 3 to 4 h markedly increased yield, whereas at higher temperatures, prolonging the extraction time had a negligible effect on extraction efficiency.

The interaction trend between pH and extraction temperature was highly consistent with that observed between extraction time and pH. At a fixed extraction duration, pH had a minor effect on the polyphenol extraction yield. Conversely, at a fixed pH, the extraction yield increased sharply with longer extraction times. Lowering the pH while extending the extraction duration further enhanced the polyphenol extraction yield.

## 3. Materials and Methods

### 3.1. Raw Material Origin and Processing

Fresh, commercially harvested Yunnan olives (*Canarium album* (Lour.) Raeusch) were purchased directly from Yunnan Kunming Agricultural Products Development Co., Ltd., Kunming, China Upon receipt, the whole fruits were washed with deionized water to remove surface impurities and subsequently dried in a forced-air oven at 60 °C until a constant weight was achieved. The dried fruits were then pulverized using a plant pulverizer (FW100, Tianjin Taisite Instrument Co., Ltd., Tianjin, China) and passed through a 60-mesh standard sieve by our research team. The resulting homogeneous olive powder was sealed in a vacuum desiccator and stored in the dark for all subsequent extractions.

### 3.2. Main Reagents and Instruments

Dried Yunnan-grown olives (commercially available from Yunnan Kunming Agricultural Products Development Co., Ltd., Kunming, China), identified as *Canarium album* (Lour.) Raeusch; gallic acid standard (≥98%, HPLC grade, Shanghai Yuanye Bio-Technology Co., Ltd., Shanghai, China); ethanol, methanol, and acetone (analytical grade, Sinopharm Chemical Reagent Co., Ltd., Shanghai, China); Folin-Ciocalteu reagent (analytical grade, Shanghai Macklin Biochemical Co., Ltd., Shanghai, China); sodium carbonate, hydrochloric acid, and sodium hydroxide (analytical grade, Sinopharm Chemical Reagent Co., Ltd., Shanghai, China).

Electronic analytical balance (FA2004N, Shanghai Mettler-Toledo Instruments Co., Ltd., Shanghai, China); thermostatic water bath shaker (SHZ-88, Jintan Dadi Automatic Instrument Factory, Jintan, China); rotary vacuum evaporator (RE-52AA, Shanghai Yarong Biochemical Instrument Factory, Shanghai, China); UV-Vis spectrophotometer (UV-2550, Shimadzu Corporation, Kyoto, Japan); pH meter (PHS-3C, Shanghai Leici Instrument Factory, Shanghai, China); high-speed refrigerated centrifuge (TGL-16M, Hunan Xiangyi Laboratory Instrument Development Co., Ltd., Changsha, China); forced-air drying oven (DHG-9070A, Shanghai Yiheng Scientific Instrument Co., Ltd., Shanghai, China); plant pulverizer (FW100, Tianjin Taisite Instrument Co., Ltd., Tianjin, China); electronic thermostatic water bath (HH-S4, Jintan Huafeng Instrument Co., Ltd., Jintan, China); pipettes (0.1–10 mL, Eppendorf AG, Hamburg, Germany); ultrapure water system (UPW-1000, Nanjing Yipu Yida Technology Development Co., Ltd., Nanjing, China); standard testing sieve (60 mesh, Shangyu Daoxu Zhangxing Sieve Factory, Shangyu, China); circulating water vacuum pump (SHB-III, Zhengzhou Greatwall Scientific Industrial and Trade Co., Ltd., Zhengzhou, China); ultrasonic cleaner (KQ-500DE, Kunshan Ultrasonic Instruments Co., Ltd., Kunshan, China).

### 3.3. Single-Factor Experiments

To determine the preliminary parameter ranges for the mathematical modeling, robust single-factor extraction experiments were conducted.

#### 3.3.1. Effect of Different Solvents

To evaluate the effect of solvent choice, four distinct and separate extraction solvent systems were prepared independently: pure water, 60% (*v*/*v*) ethanol, 60% (*v*/*v*) methanol, and 60% (*v*/*v*) acetone. For each trial, 5 g (dry basis) of the processed Yunnan olive powder was mixed with 100 mL of the respective solvent. The mixtures were shaken in a water bath at 50 °C for 3 h, filtered, and the filtrate was concentrated to dryness under vacuum at 35 °C.

#### 3.3.2. Effect of Extraction Time, Temperature, pH, Liquid-to-Solid Ratio, and Frequency

Following the identification of 60% ethanol as the optimal solvent, subsequent single-factor tests sequentially varied extraction time (1–5 h), temperature (30–80 °C), pH (2.0–7.0), liquid-to-solid ratio (5–20 mL/g), and extraction frequency (1–5 cycles). In each specific test, all non-tested parameters were held constant at the standard baseline values identified in previous steps (e.g., 60% ethanol, 3 h, 50 °C, pH 3).

### 3.4. Central Composite Design (CCD) Optimization

Based on the preliminary single-factor results, a five-level Central Composite Design (CCD) was employed to systematically evaluate the multi-factor interactions and optimize the dual-objective extraction process. Four independent variables—ethanol concentration (X_1_), extraction temperature (X_2_), extraction time (X_3_), and pH (X_4_)—were selected. The variables were coded at five levels (−2, −1, 0, +1, +2), utilizing central and “star” points to accurately map the quadratic response surfaces. The experimental factors and their corresponding actual values are detailed in Table 2. Design-Expert 7.0 was used to design the 30-run experimental protocol, which included six center-point replicates to estimate experimental error.

### 3.5. Determination of Total Polyphenol Extraction Yield

Preparation of the standard curve: Aliquots of 0.100, 0.200, 0.400, 0.600, 0.800, and 1.000 mL of a 0.200 mg/mL gallic acid standard solution were diluted to 1.000 mL. Subsequently, 1.000 mL of Folin–Ciocalteu reagent and 2.000 mL of 10% sodium carbonate solution were added sequentially, and the reaction was incubated at 25 °C for 1 h.

The absorbance (A) was measured at 750 nm using a spectrophotometer. Using the absorbance value as the ordinate and the mass of gallic acid (mg) as the abscissa, the linear regression equation was obtained as Y = 0.0215X + 0.014, with a coefficient of determination (R^2^) of 0.9989. Here, X represents the gallic acid concentration (mg/mL), and Y represents the absorbance value. The established method was validated. The analytical range exhibited excellent linearity (10–100 mg/L, R^2^ = 0.9989). Method precision was determined with intra-day relative standard deviation (RSD) < 2.5% (*n* = 6) and inter-day RSD < 3.8% (*n* = 3 days). Standard spike recovery rates ranged between 96.5% and 103.2% within the sample matrix, demonstrating the adequate precision and recovery of the total phenolic assay.

A precise amount of the polyphenol sample was weighed, dissolved in water, and diluted to a constant volume. The absorbance at 750 nm was measured and then entered into the regression equation to calculate the polyphenol content. The polyphenol extraction yield was calculated using the following equation and expressed as the polyphenol content extracted per gram of raw material:

$$\mathrm{Extraction}\;\mathrm{Yield}(\mathrm{mg}/g)=\left(C\times D\times V\times M\right)/\left(m\times W\right)$$

where C is the mass concentration of polyphenols (mg/mL);

D is the dilution factor; V is the volume (mL);

M is the total mass of polyphenols (mg);

m is the mass of polyphenols used to prepare the sample solution (mg);

W is the mass of the raw material (g).

### 3.6. Determination of Taste Capacity

Internationally, the impact of specific compound concentrations on taste is commonly evaluated using the Dot value:

Dot value=concentration of individual polyphenols in the plant extract / their respective taste thresholds.

In this study, the overall “Taste Capacity” for an extract was mathematically derived via a clear, step-by-step process. First, the concentration of each major individual polyphenolic compound (e.g., gallic acid, ellagic acid, rutin, quercetin) in the sample extract was quantitatively determined using HPLC against established standard references. Second, each specific compound’s concentration was divided by its known absolute taste threshold (i.e., established bitterness or astringency limits found in the literature) to generate its individual Dot value. A Dot value > 1 signifies a direct sensory contribution. Finally, the overall Taste Capacity of the extract was calculated by summing the individual Dot values of these major constituents [30]. It is important to note that “Taste Capacity” is not a physical constant, but rather a dimensionless empirical index used to quantitatively evaluate sensory impact. In this specific study, it reflects the combined astringent and bitter sensory load contributed by the specific polyphenolic profile of Yunnan olives. A Dot value greater than 1 (indicating that the concentration reaches the threshold) indicates that the substance may directly contribute to the taste profile, whereas a value less than 1 suggests no contribution [30]. This measurement serves as a vital bridge between chemical yield and sensory quality, making the resulting extracts highly practical for the food and tobacco industries. In this study, the overall “Taste Capacity” was defined as the aggregate sum of the Dot values of the major individual polyphenolic compounds quantified in the extracts (such as gallic acid, ellagic acid, rutin, and quercetin) using their reference bitterness/astringency thresholds reported in the literature.

Meanwhile, to facilitate comparisons with other literature data, the “concentration of polyphenols under different treatments” was converted to the content of polyphenolic substances per unit of sample dry matter (mg/g). Each sample was analyzed in triplicate. Polyphenols were quantified using their respective standard references.

All data in the charts are mean values. Prior to variance analyses (ANOVA), the assumptions of normality and homogeneity of variance for the datasets were verified using the Shapiro–Wilk test and Levene’s test, respectively. Correlation and variance analyses (ANOVA) were performed using SAS 9.2, and comparisons among treatment means were conducted using the least significant difference (LSD) method.

### 3.7. Mathematical Modeling and Simulation Analysis

Design-Expert 7.0 was used to design the response surface experimental protocols and to develop regression models for process parameter optimization. Significance testing was performed using ANOVA in OriginPro 7.0.

## 4. Conclusions

This study systematically optimized the polyphenol extraction process from Yunnan-grown olives using a combination of single-factor experiments and response surface methodology, identifying the key influencing factors and their optimal parameter combinations. By integrating “taste capacity” as a response value, this research uniquely provides an extraction protocol that satisfies both high functional yield and optimal sensory profiles. The main conclusions are as follows:

Single-factor experiments showed that solvent type, extraction time, temperature, pH, and ethanol concentration significantly affected both polyphenol extraction yield and taste capacity. Specifically, 60% ethanol was identified as the optimal extraction solvent, yielding significantly higher efficiency than water, methanol, and acetone (*p* < 0.05). Pearson correlation analysis confirmed significant relationships between structural extraction yields and subsequent taste capacity outcomes across these variables.

Acidic conditions (pH ≈ 3) effectively inhibited polyphenol oxidase activity, thereby improving polyphenol stability. A balance between extraction yield and taste capacity was achieved at an extraction time of 3 h and a temperature of 50 °C; exceeding these thresholds led to oxidative degradation of the polyphenols. To ensure a maximal polyphenol baseline for subsequent RSM optimization, an extraction frequency of 3 cycles was determined to be appropriate.

Optimization using Central Composite Design (CCD) showed that the quadratic regression models developed from the experimental data were highly significant (*p* < 0.0001). The coefficient of determination (R^2^) was 0.9433 for the extraction yield model and 0.9595 for the taste capacity model, indicating a high degree of fit and accurate predictions of actual extraction outcomes.

The impact of individual factors on extraction yield followed this order: extraction temperature > extraction time > ethanol concentration > pH. For taste capacity, the order was: extraction temperature > extraction time > pH > ethanol concentration, with extraction temperature the most critical factor.

The optimal extraction parameters for Yunnan-grown olive polyphenols were: ethanol concentration of 55.19%, extraction temperature of 50.93 °C, extraction time of 3.77 h, and pH of 2.37. Under these conditions, the experimental total polyphenol extraction yield reached 216.38 ± 3.60 mg/g, and the taste capacity was 1.27 ± 0.02, with no significant difference from the predicted values (*p* > 0.05), thereby confirming the reliability of the model.

Analysis of factor interactions revealed significant interactions among extraction temperature, ethanol concentration, and extraction time. Under high-temperature conditions, elevated ethanol concentrations exacerbated polyphenol oxidation, whereas moderate increases in extraction time compensated for insufficient dissolution at lower temperatures. The synergy between the acidic environment and ethanol effectively disrupted the bonds between polyphenols and proteins or polysaccharides, promoting efficient solute dissolution.

For successful industrial scale-up, manufacturers must account for several operational limitations. Precise thermal regulation systems are required to maintain the narrow 50–51 °C optimum safely. Moreover, while pH 2.37 prevents enzymatic degradation, it mandates the incorporation of corrosion-resistant equipment and subsequent neutralization steps using food-grade bases to ensure product compatibility. Future implementation would also benefit from systematic environmental greenness assessments. Furthermore, integrating advanced analytical workflows, such as post-column and in-source derivatization in mass spectrometry, will be vital for overcoming matrix suppression during the downstream quality control of these functional products [31].

In conclusion, the optimized process effectively balances polyphenol extraction efficiency with product taste, delivering high efficiency, stability, and cost-effectiveness. This work provides a solid scientific foundation for the industrial scale-up of Yunnan-grown olive polyphenols and for the development of related functional products.

## Figures and Tables

**Table 1:** Summary of single-factor experiment results showing optimal conditions, maximum yields, maximum taste capacity, and Pearson’s correlation coefficients.

Tested Factor	Optimal Condition	Max Yield (mg/g)	Max Taste Capacity	Pearson’s *r* (Yield vs. Taste)
Solvent type	60% Ethanol	203.7 ± 6.3	1.43 ± 0.04	0.92 *
Extraction time	3 h	203.7 ± 6.3	1.43 ± 0.04	0.88 *
Extraction temperature	50 °C	203.7 ± 6.3	1.43 ± 0.04	0.95 **
pH	3.0	203.7 ± 6.3	1.43 ± 0.04	-
Ethanol concentration	60%	203.7 ± 6.3	1.43 ± 0.04	0.91 *
Liquid-to-solid ratio	15 mL/g	203.7 ± 6.3	1.43 ± 0.04	-
Extraction frequency	3 cycles	203.7 ± 6.3	1.43 ± 0.04	-

Note: * *p* < 0.05, ** *p* < 0.01. The max yield and taste capacity values represent the optimal baseline point established across the experiments. “-” indicates data not explicitly subjected to correlation analysis.

**Table 2:** Independent variables and their actual levels used for the Central Composite Design (CCD).

Level	Factor			
	Ethanol Concentration (X_1_, %)	Extraction Temperature (X_2_, °C)	Extraction Time (X_3_, h)	Ph (X_4_)
−2	45	45	2.5	2.0
−1	50	50	3.0	2.5
0	55	55	3.5	3.0
1	60	60	4.0	3.5
2	65	65	4.5	4.0

Note: Variables $X_{1}$ through $X_{4}$ denote the coded factors utilized to represent the actual input values for the optimization modeling.

**Table 3:** Experimental design matrix and results of the CCD response surface analysis.

Run	X_1_ Ethanol Concentration (%)	X_2_ Extraction Temperature (°C)	X_3_ Extraction Time (h)	X_4_ pH	Y_1_ Extraction Rate (mg/g)	Y_2_ Taste Capacity
1	−1	−1	−1	1	191.30	0.89
2	−1	−1	1	−1	215.32	1.17
3	−1	1	−1	−1	192.32	0.88
4	−1	1	1	1	199.64	0.93
5	0	0	0	0	211.32	1.23
6	0	0	0	0	211.41	1.24
7	1	−1	−1	−1	205.97	0.96
8	1	−1	1	1	216.81	1.16
9	1	1	−1	1	200.32	0.99
10	1	1	1	−1	198.31	0.93
11	−1	−1	−1	−1	202.10	0.98
12	−1	−1	1	1	199.84	1.03
13	−1	1	−1	1	192.84	0.95
14	−1	1	1	−1	200.33	0.95
15	0	0	0	0	214.68	1.21
16	0	0	0	0	213.87	1.20
17	1	−1	−1	1	209.35	1.07
18	1	−1	1	−1	216.39	1.14
19	1	1	−1	−1	193.02	0.86
20	1	1	1	1	202.31	0.99
21	−2	0	0	0	210.64	1.13
22	0	−2	0	0	202.49	0.97
23	0	0	−2	0	192.62	0.82
24	0	0	0	−2	207.30	1.04
25	0	0	0	0	214.34	1.16
26	0	0	0	0	213.47	1.17
27	0	0	0	2	213.46	1.21
28	0	0	2	0	198.62	0.95
29	0	2	0	0	185.64	0.82
30	2	0	0	0	211.84	1.18

**Table 4:** ANOVA results for the regression Equation (1) of extraction yield.

Source	Degrees of Freedom	Sum of Squares	F-Value	*p*-Value	Significance
Model	16	2195.9488	13.5226	<0.0001	*
X_1_	1	108.8004	10.7198	0.0060	*
X_2_	1	518.0104	51.0382	<0.0001	*
X_3_	1	226.3204	22.2987	0.0004	*
X_4_	1	0.0337	0.0033	0.9549	
X_1_X_2_	1	59.6756	5.8797	0.0306	*
X_1_X_3_	1	7.9806	0.7863	0.3913	
X_2_X_3_	1	19.1406	1.8859	0.1929	
X_1_X_4_	1	108.6806	10.7080	0.0061	*
X_2_X_4_	1	70.1406	6.9108	0.0208	*
X_3_X_4_	1	9.1506	0.9016	0.3597	
X_1_X_1_	1	9.1017	0.8967	0.3609	
X_2_X_2_	1	652.1357	64.2532	<0.0001	*
X_3_X_3_	1	549.5300	54.1437	<0.0001	*
X_4_X_4_	1	17.0550	1.6804	0.2174	
Lack of Fit	10	131.2131	53.9232	0.0037	
Pure Error	3	0.7300			
Residual	13	131.9432			
Cor total	29	2327.8908			
R-squared value	0.9433	Adjusted R- squared value	0.8736		

Note: The “Significance” column uses asterisks (*) to indicate statistically significant terms (*p* < 0.05).

**Table 5:** ANOVA results for the regression Equation (2) of taste capacity.

Source	Degrees of Freedom	Sum of Squares	F-Value	*p*-Value	Significance
Model	16	0.4860	19.2637	<0.0001	*
X_1_	1	0.0074	4.6614	0.0501	*
X_2_	1	0.0620	39.3308	<0.0001	*
X_3_	1	0.0400	25.3785	0.0002	*
X_4_	1	0.0096	6.0883	0.0283	*
X_1_X_2_	1	0.0025	1.5855	0.2301	
X_1_X_3_	1	0.0001	0.0634	0.8051	
X_2_X_3_	1	0.0144	9.1324	0.0098	*
X_1_X_4_	1	0.0156	9.9093	0.0077	*
X_2_X_4_	1	0.0072	4.5821	0.0518	
X_3_X_4_	1	0.0056	3.5674	0.0814	
X_1_X_1_	1	0.0065	4.1344	0.0630	
X_2_X_2_	1	0.1774	112.4916	<0.0001	*
X_3_X_3_	1	0.1886	119.5946	<0.0001	*
X_4_X_4_	1	0.0144	9.1355	0.0098	*
Lack of Fit	10	0.0204	40.6967	0.0056	
Pure Error	3	0.0002			
Residual	13	0.0205			
Cor total	29	0.5065			
R-squared value	0.9595	Adjusted R- squared value	0.9097		

Note: The “Significance” column uses asterisks (*) to indicate statistically significant terms (*p* < 0.05).

## Data Availability

Data are contained within the article.
